# Is It Possible to Separate the Graft-Versus-Leukemia (GVL) Effect Against B Cell Acute Lymphoblastic Leukemia From Graft-Versus-Host Disease (GVHD) After Hematopoietic Cell Transplant?

**DOI:** 10.3389/fped.2022.796994

**Published:** 2022-03-24

**Authors:** Jacob Rozmus, Sima T. Bhatt, Nataliya Prokopenko Buxbaum, Geoffrey D. E. Cuvelier, Amanda M. Li, Carrie L. Kitko, Kirk R. Schultz

**Affiliations:** ^1^Division of Hematology, Oncology and Bone Marrow Transplant, Department of Pediatrics, Faculty of Medicine, British Columbia Children's Hospital, University of British Columbia, Vancouver, BC, Canada; ^2^Washington University, Saint Louis, MO, United States; ^3^Roswell Park Comprehensive Cancer Center, Department of Pediatrics, Buffalo, NY, United States; ^4^Pediatric Blood and Marrow Transplantation, Cancer Care Manitoba, University of Manitoba, Winnipeg, MB, Canada; ^5^Pediatric Hematology/Oncology Division, Vanderbilt University Medical Center, Nashville, TN, United States

**Keywords:** pediatric B-ALL, graft-versus-leukemia, graft-versus-host disease, relapse, hematopoietic cell transplantation

## Abstract

Hematopoietic cell transplant is a curative therapy for many pediatric patients with high risk acute lymphoblastic leukemia. Its therapeutic mechanism is primarily based on the generation of an alloreactive graft-versus-leukemia effect that can eliminate residual leukemia cells thus preventing relapse. However its efficacy is diminished by the concurrent emergence of harmful graft-versus-host disease disease which affects healthly tissue leading to significant morbidity and mortality. The purpose of this review is to describe the interventions that have been trialed in order to augment the beneficial graft-versus leukemia effect post-hematopoietic cell transplant while limiting the harmful consequences of graft-versus-host disease. This includes many emerging and promising strategies such as *ex vivo* and *in vivo* graft manipulation, targeted cell therapies, T-cell engagers and multiple pharmacologic interventions that stimulate specific donor effector cells.

## Introduction

Hematopoietic cell transplantation (HCT) is a curative treatment for many children with high-risk or relapsed acute lymphoblastic leukemia (ALL). Its primary benefit comes from the generation of an effective alloreactive immune response that targets leukemia cells termed the graft-versus-leukemia (GVL) effect. However, its efficacy is hampered by the simultaneous occurrence of a graft-versus-host disease (GVHD) process in which the alloreactive donor cells attack healthy tissue leading to significant non-relapse related morbidity and mortality. These two processes are closely but not invariably linked; therefore the ultimate goal of the HCT community is to develop strategies that maximize GVL while preventing GVHD.

## The Graft-Versus-Leukemia (GVL) Effect

The efficacy of HCT is based on two principles. First, the use of high-dose myeloablative conditioning before HCT reduces the risk of graft rejection facilitating full donor chimerism and directly kills leukemia cells. Several cohort studies of pediatric patients with ALL demonstrate that full donor chimerism is associated a lower risk of relapse (1–4). Secondly, the donor graft mediates a graft-versus-leukemia GVL effect, *via* alloreactive T, NK and B cells. The primary mechanisms underlying the GVL effect involves donor T-cells attacking cells expressing recipient self-antigens and NK cells attacking recipient cells lacking expression of inhibitory ligands. The therapeutic potential of donor T-cells has been surmised from: (1) clinical studies demonstrating that recipients of syngeneic HCT have a higher incidence of relapse with a reduction in GVHD (5); (2) increased relapse risk associated with extensive *ex vivo* T-cell depleted donor grafts (6); (3) cure of patients who underwent non-myeloablative and reduced intensity HCT where the conditioning would provide minimal anti-leukemia effect; (4) the successful use of donor lymphocyte infusions post-HCT in treating relapse and most importantly (5) a decreased risk of relapse associated with grade I-II acute GVHD (aGVHD) and chronic GVHD (cGVHD).

NK cells are part of the innate immune system, kill cancer cells without prior sensitization and have demonstrated an important role in GVL, particularly in T-cell depleted haploidentical HCT. Their function is dictated by a range of inhibitory and activating cell surface receptors including killer cell immunoglobulin-like receptor (KIR) and C-type lectin receptors. Major ligands for KIR are MHC class I molecules that define “immune self.” Specific MHC-I-binding inhibitory KIR receptors on NK cells prevent these cells from attacking normal cells that have the matching MHC-I surface molecules. This allows donor NK cells to preferentially attack abnormal cells that have down-regulated surface MHC-I molecules, an event that occurs in cancer and virus-infected cells termed missing self-recognition, or recipient cells with incompatible inhibitory KIR ligands arising from HLA-disparate transplants. To date, KIR-ligand mismatch in the graft-versus-host direction has only been shown to be associated with a significant reduction in relapse in acute myeloid leukemia, primarily in the setting of T-cell depleted haploidentical transplantation (7, 8). A large analysis of donor KIR in the pediatric acute leukemia population did not support the use of KIR in the selection of unrelated donors for children undergoing T-replete transplantation (7).

B cells may also play an important role in GVL. It is well described that both major histocompatibility complex and minor histocompatibility antigens can elicit B-cell antibody responses. The presence of circulating HLA donor-specific antibodies increases the risk of primary graft failure in HLA-mismatched allografts (9). It is possible that alloantibodies may also play a role in disease remission. Studies have shown a highly significant association between H-Y antibodies and decreased relapse in male patients with female donors (10, 11). However, this effect is also directly related to increased rates of chronic GVHD.

The most serious consequence of the GVL effect is the potential risk of both acute and chronic GVHD, where alloreactive T-cells attack recipient antigens expressed on healthy tissue, in addition to those restricted to hematopoietic lineages containing the malignant cells. The ultimate goal of the GVL effect is to direct donor T-cells to attack antigens unique to leukemia cells whilst sparing other recipient antigens that are ubiquitously expressed.

## Impact of GVHD on all Relapse

The first description of the GVL effect was in ALL, where post-HCT recipients with moderate to severe chronic GVHD (cGVHD) were significantly less likely to relapse (12). The most recent comprehensive analysis to evaluate the relative roles of both aGVHD and cGVHD on the GVL effect following HCT for ALL was performed by the Center for International Bone Marrow Transplant Research (CIBMTR) on 5,215 transplant recipients (13). Three cohorts were assessed: 2,593 adults in first or second complete remission (CR1/CR2), 1,619 pediatric patients in CR1/CR2, and 1,003 patients with advanced (CR≥3 or active disease) ALL. For children with ALL in CR1/CR2, aGVHD of any grade was associated with lower risk of relapse compared to no GVHD, however, grade III-IV aGVHD with or without cGVHD was associated with increased non-relapse mortality (NRM), resulting in decreased disease free and overall survival. For pediatric and adult patients with advanced ALL, development of grades III-IV aGVHD or *de novo* cGVHD was associated with lower relapse rates, however increased NRM resulted in significantly worse DFS, compared to significantly improved OS among patients with cGVHD with or without lower grade aGVHD.

The relative importance of aGVHD for children with ALL was confirmed by the Westhofen Intercontinental Group (*N* = 616) analysis from both European and North American patient cohorts (14). This analysis focused on the role of both minimal residual disease (MRD) and aGVHD on event-free survival and relapse rates. Patients with and without MRD had a three-fold decrease in relapse rates post-HCT if they developed aGVHD. Importantly, as in the CIBMTR analysis, aGVHD grade IV resulted in poorer outcome due higher non-relapse mortality, negating any benefit of GVL. This study did not assess the impact of cGVHD on relapse. The occurrence of aGVHD was also been found to be important in defining relapse risk of a pre-HCT next generation sequencing (NGS)-MRD positive population of pediatric patients with B-ALL. Among 19 pre-HCT MRD positive patients, the estimated 2-year relapse probabilities were 73% for patients with no aGVHD by day +55 and 17% for those who experienced aGVHD by day +55 (*P* = 0.02) (15). An earlier Italian study that evaluated the impact of cGVHD on pediatric HCT outcomes included 450 patients with malignancy, including 268 with ALL (16). In the cohort of patients with malignant disease, cGVHD was associated with decreased risk of relapse, and this effect seemed strongest in patients with ALL. When the entire cohort was analyzed, no impact of aGVHD grades 0-I vs. II-IV was observed on the risk of relapse.

While there is evidence that both aGVHD and cGVHD contribute to the GVL effect in children with ALL, it is difficult to translate this understanding into actionable clinical interventions for any given patient because despite both acute and chronic GVHD being associated with GVL, severe GVHD results in increased NRM and decreased survival. New post-HCT strategies are needed to further augment GVL with minimal to no acute or chronic GVHD.

## Interventions to Promote GVL

**Figure 1:**
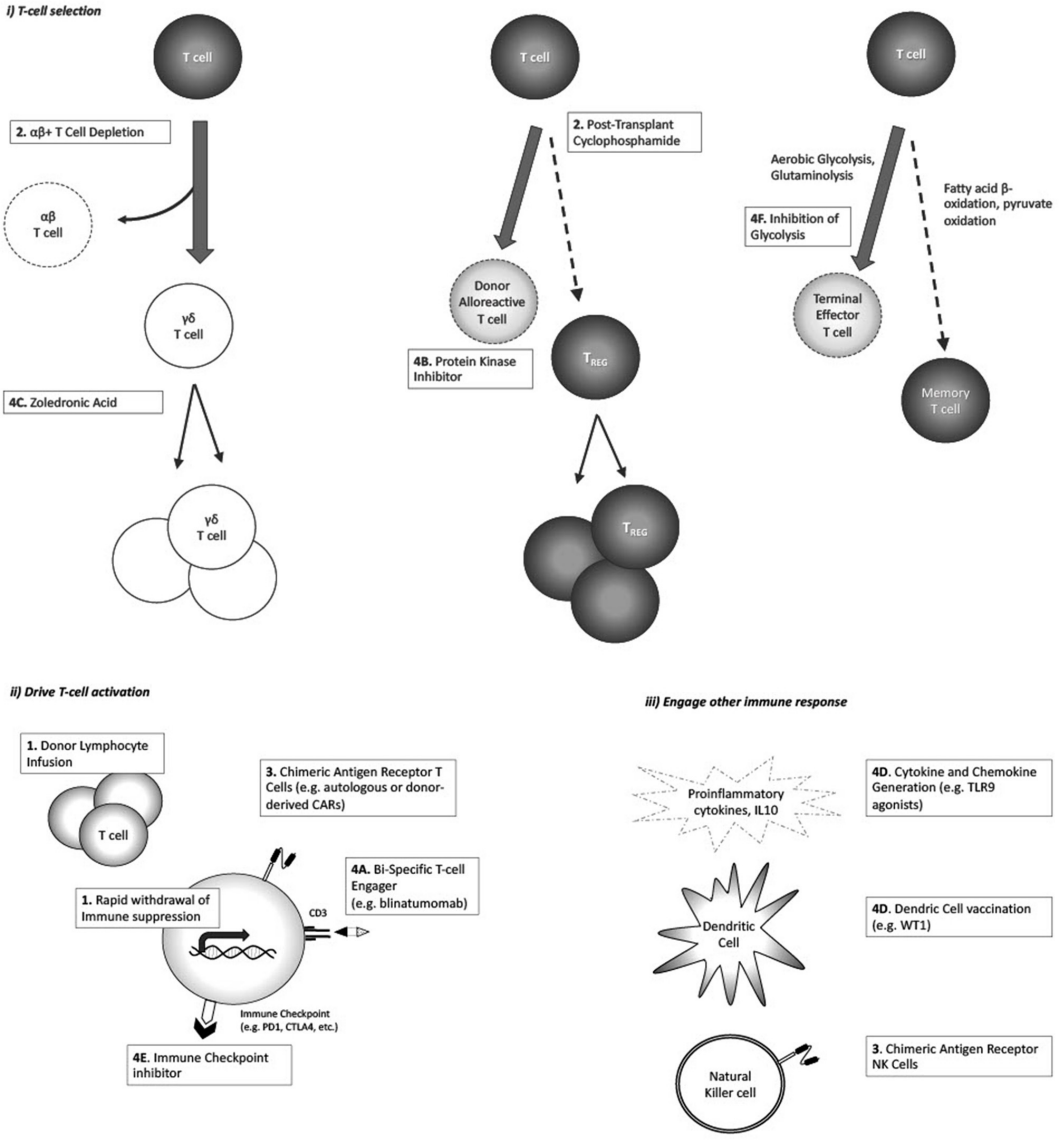
Strategies postulated to augment GVL response, and/or enhance GVL without increasing GvHD: **(i)** Enhance selective T-cell populations; **(ii)** Drive T-cell activation against tumor antigen; **(iii)** Engage non-T-cell immune responses.

GVL and GVHD have similar but not identical targets. The goal for the HSCT field remains the enhancement of the GVL effect while limiting or eliminating GVHD. The purpose of this review is to describe several strategies that have been undertaken in an attempt to tip the alloimmune balance toward GVL (Table 1; Figure 1).

**Table 1:** Cellular and pharmacologic approaches to modify graft vs. leukemia effect post-HCT for ALL.

**Intervention**	**Proposed mechanism of action**	**Outcome**	**Limitations**	**Active pediatric clinical trials**	**References**
Donor lymphocyte infusion	Enhance GVL^*^	20–70% RR^#^	Severe GVHD^&^	NCT05009719 NCT03297528	(17–23)
CAR-T	Antigen directed genetically modified autologous T-cell immunotherapy	50–80% RR^#^	Antigen negative relapse, CRS^∧^, neurotoxicity	NCT04544592 NCT03853616 NCT04016129 NCT04276870 NCT04173988 NCT02650414	(24–27)
NK-CAR	Antigen directed NK-CAR	73% RR^#^ in adult Phase I/II study	N/A	NCT03056339	(28, 29)
Blinatumomab	CD19 BITE that may redirect an otherwise unengaged polyclonal donor T-cells to attack CD19+ ALL cells	N/A	Many upfront and bridging therapies are incorporating this agent, potentially diminishing its utility	NCT04044560 NCT02790515 NCT03849651	(30, 31)
mTOR inhibitor	Decrease Grade 2–4 aGVHD^&^ and relapse	Decreased aGVHD^&^ but did not improve survival	Increase of transplant related morbidity	No active trials	(32)
Zoledronic Acid	Induce differentiation and increase cytotoxicity of the Vδ2 subset	N/A	N/A	NCT02508038	(33, 34)
Vaccines	Expand donor derived leukemic specific T-cells	N/A	N/A	NCT03559413	(35–42)
Immune Checkpoint Inhibitors	Inhibit the immune regulatory molecules expressed on leukemic cells	0–70% RR^#^	Severe GVHD^&^	NCT03286114 NCT03588936 NCT03146468 NCT01822509	(43–48)

* *Graft vs. leukemia*,

# *Response Rate*,

& *Graft vs. host disease*,

∧ *Cytokine Release Syndrome. Cellular therapy and Pharmacologic Interventions to Promote GVL*.

### 1. Early Withdrawal of Immunosuppression and Donor Lymphocyte Infusions

Early withdrawal of immune suppression and donor lymphocyte infusions are commonly used strategies for relapse post-HCT, although there is a lack of published evidence as to their efficacy in pediatric ALL. Clinically meaningful effects related to donor lymphocyte infusion (DLI) have been described in chronic malignancies such as follicular, mantle cell, small lymphocytic, Hodgkins lymphoma, chronic myeloid leukemia and myeloma (17, 18, 49, 50). Withdrawal of GVHD prophylaxis to reduce relapse risk is an intervention that can only be done early post-HCT. There is broad consensus, despite the absence of published standardized pediatric guidelines, that the duration of GVHD prophylaxis after HSCT for malignant disease should be 180 days (19). In a survey of European pediatric HSCT centers, the duration of GVHD prophylaxis was shortened to 60–120 days post-HSCT if the relapse risk was categorized as high (20). It is reasonable to define fast withdrawal of immunosuppression (FWI) as occurring prior to 60 days post-HSCT. Therefore, FWI really only applies to early relapse, which implies high-risk disease and may be expected therefore to have limited benefit. Immunosuppression is usually withdrawn as a prelude to another interventions, such as DLI. DLI used alone or in combination with additional agents has been employed to enhance GVL in the setting of relapse after HCT. However, the use of DLI is limited by development of GVHD. Data for DLI alone have largely come from adult studies that demonstrate minimal efficacy in lymphoid malignancies with a high risk of GVHD (21, 22). A single center retrospective review of 30 pediatric patients (myeloid, *n* = 23; lymphoid (ALL), n=7) receiving DLI for relapse after HCT reported a 5-year disease free survival of 32% for all patients. The lymphoid group had a 5-year survival rate at 71±17% compared to the myeloid group at 22 ± 9%. In the case of HLA-matched donors the initial median CD3/kg doses were 1–5 × 10^7^/kg with escalation to 8 × 10^7^/kg for subsequent doses. For HLA-mismatched donors, the initial median CD3/kg dose was lower at 5 × 10^5^/kg with subsequent infusions escalated to median of 5 × 10^6^/kg. In this retrospective study, the development of GVHD did not affect overall survival (23). In an attempt to improve effectiveness while minimizing toxicity, several centers are trialing dose escalating schedules of DLI or repetitive administration of low dose DLI (51, 52).

An alternate strategy would be to pre-emptively withdraw immune suppression combined with DLI early in select patients based on high-risk features such as pre- and post-HCT MRD or mixed chimerism post-HCT. One study of pediatric patients with mixed chimerism undergoing immune withdrawal and DLI included 17 patients with ALL out of total of 43. The first step was FWI with evidence of mixed chimerism post-HCT, followed by increasing DLI doses if mixed chimerism persisted after withdrawal of immunosuppression. Twenty-six (60%) patients with mixed chimerism were assigned to immune withdrawal, which started at a median of 49 days (range, 35 to 85 days) after HCT. Fourteen patients proceeded to DLI after withdrawal at a median of 118 days (range, 85 to 194 days). The DLI dose for matched donor transplant recipients was 1 × 10^6^ CD3/kg escalating to 1 x 10^8^/kg; 1 x 10^5^/kg to 1 x 10^7^/kg for mismatched donor transplant recipients. The intervention cohort had a similar 2-year event-free survival (EFS) [73; 95% confidence interval (CI), 55 to 91%] compared with patients who achieved full donor chimerism spontaneously (83; 95% CI, 62 to 100%). There were no late relapses in the observation group with full donor chimerism, but 50% of all relapses in the intervention group occurred more than 2 years after transplantation and their EFS declined to 55% (95% CI, 34 to 76%) at 42 (SD, 11) months. Nineteen percentage of patients undergoing the intervention developed GVHD. Consistent with previous observations, the development of cGVHD was protective against relapse (53).

There are a number of strategies being investigated to reduce the risk of GVHD associated with DLI while maintaining GVL including depleting the DLI product of alloreactive T-cells by *ex vivo* photodepletion and inserting an inducible suicide gene in donor lymphocytes so that they can be eliminated when GVHD occurs (54, 55).

### 2. Post-HCT Cyclophosphamide (PTCy) and TCR αβ^+^/CD19^+^ Depletion

The last decade has seen a rise in the use of HLA haploidentical allogeneic HCT for pediatric and adult ALL. Several *T*-cell replete and T-cell depleted haploidentical transplant strategies are currently used to overcome the barriers of GVHD and graft failure. In T-cell replete haploidentical HCT, which involves the infusion of unmanipulated stem cell product followed by *in vivo* depletion of alloreactive T-cells, the use of post-transplant cyclophosphamide (PTCy) has rapidly increased due to its simplicity and efficacy. In terms of *ex vivo* T-cell depletion strategies, TCR αβ^+^/CD19^+^ depletion is increasingly being used as it maintains NK cell alloreactivity while limiting GVHD. Given excellent outcomes in the haploidentical setting, both of these approaches are increasingly being explored in the matched unrelated and sibling donor setting. An interesting observation seen with both approaches has been acceptable leukemia free survival but with relatively lower incidences of severe grades III-IV aGVHD and cGVHD (56–60). This suggests preservation of a GVL effect but with diminution (although not complete abrogation) of GVHD.

Initial models suggested the mechanism by which PTCy induced immune tolerance involved the selective killing of highly proliferative host-alloreactive donor T-cells after cyclophosphamide infusion on day +3. Longer-term immune tolerance induction then occurred through intrathymic clonal deletion of donor HSC-derived anti-host T-cells (61). Clinical observation, however, has shown that grade II aGVHD is still frequent after PTCy (20–40% range) and when present, improves progression free survival in hematologic malignancies (62–65). This suggests that alloreactive donor T-cells capable of inducing both GvHD and GVL persist after cyclophosphamide. Murine models have provided further insight into PTCy mechanisms of action, raising questions about the original mechanisms believed to underly PTCy immune tolerance (66– 69). More contemporary working models of PTCy suggest: (1) CD4^+^CD25^+^Foxp3^+^ regulatory T-cells (T_REGS_) are imperative in the early prevention of GVHD after PTCy, helping to control alloreactive effector donor T-cells. High levels of aldehyde dehydrogenase in T_REGS_, the major detoxifying enzyme for cyclophosphamide, prevents their killing to the same extent as effector T-cells after PTCy, allowing early and expanded post-transplant T_REG_ reconstitution despite CD4 lymphopenia (24, 25). Preferential T_REG_ reconstitution following PTCy has also been demonstrated to be time and dose dependent in an MHC-haploidentical murine mouse model, with greatest impact on T_REG_ reconstitution when cyclophosphamide is given on day +4 (24). The suppressive effects of T_REGS_ appear to constrain new host-alloreactive effector T-cells both early and late after PTCy HCT, thus keeping severe aGVHD and cGVHD in check (27). (2) Highly proliferative host-alloreactive donor CD8^+^ effector T-cells are not eliminated after PTCy, but are intact and made functionally impaired, reducing their ability to cause GvHD (26). This impairment is likely related to both direct effects of PTCy (immediate) and preferential reconstitution of T_REGS_ (late). (3) Host-alloreactive donor CD4+ effector T-cells are killed and have reduced proliferation after PTCy, a phenomenon that appears important in preventing aGVHD. Providing PTCy in either reduced dose or on different days increases CD4+ effector T-cells and results in rapid death in an MHC-haploidentical acute GVHD mouse model (28). Our understanding of how PTCy modulates immune tolerance, while still allowing GVL to develop and prevent leukemia relapse, remains incomplete. The impact of other concurrently administered GVHD prophylaxis medications used in clinical practice, such as calcineurin inhibitors, mycophenolate mofetil and anti-thymocyte globulin and the selective infusion of other effector cells on GVHD and GVL after PTCy, require further investigation. For example, there is a phase II pilot study investigating whether the infusion of *ex-vivo* expanded natural killer cell infusions in children wih myeloid leukemia receiving HLA-haploidentical HCT with PTCy decreases relapse rates and infectious complicaitons without increasing GVHD (NCT#04836390). In addition, recent registry data suggest that HLA matching still matters with PTCy, with lower rates of grade III-IV aGVHD in adults with acute leukemia following matched unrelated donor compared to HLA-haploidentical transplant when a common PTCy backbone was compared (25).

By comparison, *ex-vivo* graft manipulation to remove GVHD causing TCRαβ^+^ T-cells (TCRαβ^+^/CD19^+^ depletion) has also gained traction in pediatric acute leukemia to overcome HLA disparity (60). The selective removal of most TCRαβ^+^ T-cells appears to reduce both aGVHD and cGVHD, while maintaining NK cells and TCRγδ^+^ T-cells that have less host alloreactivity but are able to mediate GVL (26). A number of potential mechanisms exist by which TCRγδ^+^ T-cells and NK cells mediate GVL, including the shared presence of activating receptors (e.g., NKG2D) that are independent of tumor antigen recognition in the context of MHC, thus able to bypass tumor escape through MHC class I downregulation (27). NK alloreactivity through killer immunoglobulin-like receptor (KIR) recognition of MHC class I KIR/KIR-ligand mismatch in a donor-versus-recipient direction has been also purported to exert a GVL effect although this has not been seen to impact leukemia-free survival in one large acute leukemia study in children (69).

### 3. Non-HCT Cellular Therapy

Some anti-leukemic strategies used in the pre-HCT setting are also being used in the setting of relapse post-HCT, including CAR-T and CAR-NK (29). However, a significant proportion of patients relapse after cellular immunotherapy without HCT consolidation, suggesting that lasting GVL may require immune responses that are oligoclonal. Therapeutic efficacy has been observed with the use of tisagenlecleucel, a CD19-directed CAR-T therapy that is FDA approved for the treatment of relapsed, refractory pre-B ALL and has demonstrated durable remissions in patients that relapse after transplant (70). However, there are limitations to this approach, including the ability to generate autologous CAR-T cells from patients that may be lymphopenic after transplant, time to manufacture product, and antigen escape. To address some of these barriers, donor-derived CAR-T cells have been successfully tested by several groups, with low risk of GHVD and response rates ranging from 50 to 80% (30, 31). Donor-derived virus-specific T-cells, engineered to express CD19. CAR, have also demonstrated antitumor activity early post-HCT for relapsed B-cell malignancies (71).

Other groups have explored the utility of CAR-NK cells to avoid the CAR-T related toxicities of cytokine release syndrome (CRS), neurotoxicity, and prolonged B-cell aplasia. Herrera et al. explored the utility of CAR-NK cells obtained from peripheral blood or cord blood as a potential candidate for allogeneic therapy (72). Additionally, due to the shorter lifespan of NK cells, they hypothesized that B-cell aplasia may not be as prolonged as typically seen after CAR-T cell infusion. Indeed, a recent Phase I/II trial of adult patients with lymphoid malignancy demonstrated a 73% response rate in 11 patients treated with CAR-NK cells with no patients developing CRS, neurotoxicity, or GVHD (73).

### 4. Pharmacologic Agents During/After HCT That Stimulate Donor Immune Effector Cells

#### A. Blinatumomab

Blinatumomab is a bispecific T-cell engager (BiTE) consisting of CD3 and CD19 single-chain variable regions that allow cytotoxic T-cells to specifically target and lyse CD19-positive cells, i.e., malignant and normal B cells. Unlike more traditional antibody-drug conjugate such as inotuzumab, BiTEs form a link between T-cells and leukemia cells. In the post-HCT setting, it is hypothesized that blinatumomab could redirect an otherwise unengaged polyclonal donor T-cells to attack CD19^+^ ALL cells. Blinatumomab could serve as an adjuvant for the GVL effect by redirecting donor T-cells toward malignant lymphoblasts. This approach could be especially beneficial in patients with genomic loss of HLA expression on malignant cells post-HCT, which occurs in up to 30% of haploidentical HCTs (74, 75). This renders them invisible to donor T-cells attacking minor histocompatibility antigens. However, the usage of blinatumomab post-HCT may be limited by its' increased use as a bridging therapy pre-HCT to achieve MRD negativity, which unfortunately leads to the downregulation of CD19 expression on leukemic cells in a significant proportion, up to 25%, of cases (76). The role of blinatumomab post-HCT is currently being evaluated in a number of single arm, open label studies, including a multi-centre Canadian phase II study using blinatumomab for treatment of detectable MRD in the first year following allogeneic HCT for patients with B-ALL (NCT#04044560), as well as studies examining TCRαβ and CD45RA depleted haploidentical HCT followed by blinatumomab in the early post-engraftment period and TCRαβ/CD19-depleted haploidentical HCT followed by CD45RA-depleted DLI and blinatumomab in pediatric patients with CD19^+^ malignancy (NCT#02790515 and NCT#03849651).

#### B. Protein Kinase Inhibitors

Some pharmacologic strategies may produce a synergistic effect of GVHD suppression while generating GVL, for instance tyrosine kinase inhibitors (TKIs). Whether the use of TKIs in GVHD results in improved GVL or lower relapse rates has not been elucidated, but could be anticipated given that tyrosine kinases, including Syk, Btk, and Itk, are key molecular targets in both, hematologic malignancies (32) and in alloreactive T-and B cells in GVHD (77). Given expanding therapeutic use of TKIs for GVHD (33, 34) the potential impact on GVL could be evaluated. Similarly JAK inhibitors, including ruxolitinib (35, 36) and itacitinib, that are either approved or undergoing clinical testing for GVHD, respectively, have the potential to impact on GVL (less clear whether positively or negatively), which warrants further study. In a randomized phase 3 COG/PBMTC trial, the addition of sirolimus, an mTOR inhibitor, to tacrolimus/methotrexate GVHD prophylaxis in children with ALL decreased grade 2–4 aGVHD but did not improve survival as the occurrence of grades 1–3 aGVHD showed a trend toward decreased relapse and improved EFS (37).

#### C. Zoledronic Acid

A previous study showed that subsets of γδ T-cells taken from children following αβ^+^T cell and CD19^+^ B cell depleted HLA-haploidentical HCT, display a cytotoxic phenotype and degranulate when challenged with lymphoid leukemic blasts. These cells have been shown to expand *in vitro* following exposure to zoledronic acid and are able to efficiently lyse primary lymphoid blasts (38). Zoledronic acid infusions were shown to induce differentiation and increase cytotoxicity of the Vδ2 subset *in vivo* (39). This led to an open-label, feasibility, proof-of-principle study in 46 children on the use of zoledronic acid to enhance TCRγδ+ lymphocyte function after TCRαβ/CD19-cell depleted haploidentical HCT (40). However, due to the limited number of patients enrolled and events observed, it was not possible to draw any firm conclusions on reduction in relapse. Further investigation is needed and a non-randomized prospective trial is ongoing (NCT02508038).

#### D. Vaccines With Immune Adjuvants

Another active area of research is the use of vaccines in the immediate post-HCT setting to expand donor derived leukemic specific T-cells while taking advantage of the strong lymphopenia-triggered drive for lymphocyte expansion post-HCT (41). This immune response can be further boosted using adjuvants as TLR agonists and exogenous cytokines which induce expression of effector cytokines and chemokines, recruit and activate immune cells and enhance antigen uptake and presentation (42, 43, 78). In murine models, treatment with synthetic oligodeoxynucleotides, containing unmethylated cytosine-phosphate-guanosine (CpG) motifs that bind TLR9, enhanced GVL effects without worsening GVHD (44–46). CpG stimulation of primary precursor B-ALL samples induced the release of proinflammatory cytokines and IL-10 and shifted allogeneic T-cell responses toward a Th1 pattern of cytokine production (47).

There have also been pilot trials assessing the feasibility of a WT1 peptide-loaded donor-derived dendritic cell (DC) vaccine given with DLI to enhance and direct the GVL effect (48, 79).

#### E. Immune Checkpoint Inhibitors

PD-1 blockade has been used in patients with refractory/relapsed B-cell ALL with CAR T-cell loss or insufficient response to anti-CD19 CAR T-cell therapy (80). There are only 3 reported cases of immune checkpoint inhibitor (CPI) therapy being used in adult patients with relapsed ALL post-HCT (81, 82). Only one patient experienced a therapeutic response. Risk of GVHD with CPI exposure is around 23% if given to a post-allo-HCT population (83). About 14% of cases were reported with aGVHD and 9% of patients suffered from cGVHD. Fatal GvHD has been reported in relapsed lymphoma post-HCT (84, 85). The studies so far in other hematological malignancies suggest the frequency and severity of immune-related adverse events and GVHD are higher in anti-PD-1 treated patients than in anti-CTLA-4 treated patients in the post-HCT setting (86). It remains to be seen whether a particular dosage or proper timing of CPI can increase efficacy while lowering the risk of GVHD. There are several open phase I studies investigating the augmentation of the GVL effect *via* checkpoint blockade in adult patients with relapsed ALL post-HCT (NCT03286114, NCT03588936, NCT03146468, NCT01822509).

#### F. Targeting Alloreactive T-Cell Metabolism

It has been proposed that T-cells follow 2 different differentiation pathways post-HCT based on their metabolic activity. Some activated naïve T-cells rapidly increase their metabolic activity by switching from fatty acid β-oxidation and pyruvate oxidation *via* the tricarboxylic (TCA) cycle to aerobic glycolysis and glutaminolysis (87–89). This population is driven toward a terminally differentiated effector state that is associated with limited lifespan, diminished replicative potential, and ultimately earlier cell senescence. It is hypothesized that these T-cells are associated with GVHD. In contrast, lower metabolism rates during T-cell activation may favor the formation of longer-lived memory T-cells that enhance the GVL effect (90). Therefore, it is possible that inhibition of glycolysis could inhibit GVHD driven by hypermetabolic terminally differentiated effector T-cells while preserving a GVL effect reliant on long term memory T-cells.

## Concluding Remarks

The development of better strategies to preferentially augment GVL will only come from the further elucidation of the mechanisms underlying the alloreactive immune responses post-HCT. It is clear that the GVL effect is intimately related to GVHD but emerging evidence from laboratory models and translational research suggest there are differential mechanisms which can be exploited. By isolating and amplifying those immune processes that specifically target leukemia cells we can tip the balance toward a beneficial alloreactivity while limiting toxicity. The ultimate goal of fully separating GVL from GVHD has yet to be realized.

## Author Contributions

JR compiled the final draft of the manuscript. All authors wrote sections of the manuscript, contributed to manuscript revision, read, and approved the submitted version.

## Funding

This study received funding from the St. Anna Children's Cancer Research Institute, Vienna, Austria. The funders were not involved in the study design, collection, analysis, interpretation of data, the writing of this article, or the decision to submit it for publication.

## Conflict of Interest

The authors declare that the research was conducted in the absence of any commercial or financial relationships that could be construed as a potential conflict of interest.

## Publisher's Note

All claims expressed in this article are solely those of the authors and do not necessarily represent those of their affiliated organizations, or those of the publisher, the editors and the reviewers. Any product that may be evaluated in this article, or claim that may be made by its manufacturer, is not guaranteed or endorsed by the publisher.
